# Transforming care: a study on physicians’ awareness, attitudes, and barriers to pressure injury prevention in medical wards in Jordan

**DOI:** 10.3389/fpubh.2025.1582074

**Published:** 2025-05-14

**Authors:** Enas A. Assaf, Rahaf Alkhresheh, Haleama Al Sabbah, Hekmat Al-Akash

**Affiliations:** ^1^College of Nursing, Khamis Mushait, King Khalid University, Khamis Mushait, Saudi Arabia; ^2^Faculty of Nursing, Applied Science Private University, Amman, Jordan; ^3^College of Health Sciences, Abu Dhabi University, Abu Dhabi, United Arab Emirates

**Keywords:** pressure injury, medical ward, Jordan, physicians, knowledge, attitudes, barriers

## Abstract

**Background:**

Pressure injury is a significant global concern, with rising prevalence and substantial direct and indirect costs. A multidisciplinary approach involving nurses and other healthcare team members, particularly physicians, is essential for comprehensively addressing pressure injury (PI) in medical wards. Therefore, this study aims to assess the knowledge, attitudes, and barriers among physicians working in Jordanian general medical wards toward the prevention of pressure injury.

**Method:**

A descriptive correlational cross-sectional design was employed, utilizing a convenience sample of 73 physicians from the three largest governmental hospitals in Jordan, representing the country’s most populated regions. A self-administered questionnaire measuring knowledge, attitudes, and barriers was distributed among the study population. Descriptive statistics, including mean (M) and standard deviation (SD), were used to analyze the total scores, while a linear multiple regression model was applied to identify factors influencing knowledge, attitudes, and barriers toward pressure injury prevention in Jordanian medical wards.

**Results:**

The study found that physicians demonstrated good knowledge but exhibited weak attitudes and recognized significant barriers toward pressure injury prevention. Higher income was associated with significant differences in knowledge and attitude scores (*p* < 0.00 and *p* < 0.05, respectively), while the universal guidelines correlated with significant differences in knowledge scores (*p* < 0.048). Additionally, recognized barriers showed significant differences among those using guidelines and perceived unproportioned staff (*p* < 0.041, *p* < 0.03) respectively.

**Conclusion:**

Physicians recognized significant barriers that negatively influenced their attitudes toward implementing preventive measures in medical wards. Strengthening the multidisciplinary team approach in medical wards by clarifying roles and responsibilities could enable physicians to more effectively participate in PI prevention efforts.

## Introduction

Pressure injury (PI) is defined as localized damage caused by pressure, or pressure combined with shear, to the skin and/or underlying tissue, typically over a bony prominence (1). Despite technological advancements and preventive measures, pressure injury (PI) remains a serious global health concern (2). The global prevalence of PI was 12.8%, with rates of 14.5% in Europe, 13.6% in North America, 12.7% in South America, 3% in Asia, 12.6% in the Middle East, and 9% in Australia between 2008 and 2018 (3, 4). PI imposes substantial economic burdens on healthcare systems and society. Direct costs associated with treating PI include expenses for wound care supplies, medications, and specialized interventions such as surgical debridement or skin grafting (5). Indirect costs arise from prolonged hospital stays, increased utilization of healthcare resources, and potential litigation (6). In the United States alone, the annual cost of treating PI exceeds $11 billion, underscoring the financial impact of this preventable condition (2).

Knowledge plays a crucial role in preventing PI among healthcare providers (7). When healthcare providers have a deep understanding of risk factors, preventive measures, and proper protocols related to PI, they are better equipped to identify early signs, implement preventive strategies, and provide appropriate care to at-risk patients (8). Attitudes also significantly influence PI prevention among healthcare providers (9). Positive attitudes toward patient care and adherence to protocols significantly influence the effectiveness of PI prevention efforts (10).

Barriers can significantly impact the prevention of PI among healthcare providers (11). When healthcare professionals encounter barriers such as lack of resources, time constraints, inadequate staffing, or limited knowledge and training, their ability to effectively implement preventive measures is hindered (12, 13).

Traditionally, pressure injury prevention has been the responsibility of nurses. However, a multidisciplinary approach involving nurses and other healthcare team members, particularly physicians, is essential for comprehensively addressing PI in medical wards (13–15). A study in Africa reported point prevalence rates of PI ranging from 3.4 to 18.6% in medical, surgical, and other general hospital units, with a pooled prevalence of 11% in grade II–IV hospitals and 44% in spinal injury units (16). In Kuwait, a study found that 203 out of 1,186 hospitalized patients (17.1%) had PI across 54 medical wards in the country’s seven leading general hospitals (17). A recent study in Jordan reported a PI prevalence of 29% in intensive care units and 12% overall in medical contexts (3).

In Jordanian general medical wards, the prioritization of pressure injury (PI) prevention may be hindered by competing priorities, limited resources, and a lower perceived risk compared to intensive care units (18). The workload in medical wards often involves large patient ratios and limited resources, leading to a reactive rather than proactive approach to PI management, where special interventions are provided only after injuries occur (19).

Physicians should address conditions that contribute to increased PI occurrences by prescribing appropriate treatments and medications (4, 20). A study evaluating the perspectives of physicians on PI prevention and management at King Fahad Medical City in Saudi Arabia found that doctors displayed a positive attitude, with an average mean score of 42.35 ± 4.65. However, no significant differences in attitude scores were found among subgroups (4, 20). Another study indicated that work experience, PI prevention-related knowledge, and attitudes significantly impacted care performance (21). A previous study in New York reported limited involvement of physicians in wound care, particularly in PI prevention (22).

While several studies in Jordan have assessed the involvement of nurses and other healthcare providers in PI prevention, none have specifically evaluated the knowledge, attitudes, and barriers faced by physicians working in medical wards. Given the unique health conditions and workload concerns in these settings, this study aims to assess the knowledge, attitudes, and main barriers of physicians working in general medical wards in Jordan toward the prevention of PI using a cross-sectional correlational design.

## Materials and methods

### Study design, sampling, and setting

A descriptive correlational cross-sectional design was employed, involving 73 physicians working in general medical wards at the three largest governmental hospitals in Amman, the capital of Jordan, in Irbid (the northern city of Jordan), and in the southern region of Al-Karak. The sample was selected using the convenience sampling method from physicians. Convenience sampling is “the use of the most readily accessible healthcare providers as subjects in a study” (23). The abovementioned hospitals were chosen due to the fact that they received patients from various socioeconomic backgrounds and patients from all governorates and neighboring regions. Moreover, all hospitals follow the same Ministry of Health guidelines and policies. The inclusion criteria were all physicians in residency programs in the medical wards, excluding the specialists and the physicians in administrative positions.

The sample size refers to the number of participants required to attain statistically reliable outcomes (24). It constituted a crucial element of research design, influencing the validity of the study and the relevance of its findings. The researchers utilized G*power software to calculate the appropriate sample size for the study. With an alpha level of 0.05, a power of 95%, and a mild effect size of 0.15 for a two-tailed test, Pearson’s correlation coefficient was selected as the statistical test. This test provided a standardized measure of the linear relationship between the variables.

### Research instrument

The study used a self-administered questionnaire (25). The questionnaire consisted of four parts, with the first part collecting demographic data and factors related to healthcare providers in the internal medicine departments of Jordanian hospitals. This included information on gender, age, marital status, qualifications, monthly income, work experience, training on pressure injury (PI), use of pressure injury guidelines, and perceived unproportioned staff. The tool showed good convergent validity, a high degree of internal consistency, and a reliability coefficient of 0.88 (25).

The second part involved testing knowledge using the adapted, validated, and reliable questionnaire. It consisted of 18 knowledge questions, each with three response options on a scale from 0 to 2 (0 = I do not know, 1 = False, and 2 = True). The tool showed good convergent validity, a high degree of internal consistency, and a reliability coefficient of 0.88 (25).

The third part of the questionnaire assessed the attitudes of healthcare providers using the Pressure Injury Attitude Questionnaire, which consists of 11 questions with a 5-point Likert scale: 5 for “5 strongly agree,” 4 for Agree,” 3 for “neither agree nor disagree,” 2 for “disagree,” and 1 for “strongly disagree.” Scores greater than or equal to the mean of attitude-related questions were considered a good attitude, while scores below the mean were considered a poor attitude. The tool showed internal consistency, with a reliability coefficient (Cronbach’s *α*) of 0.76 (26).

The fourth part of the data collection tool in the questionnaire comprised 12 closed-ended questions (‘Yes’ or ‘No’ response) to identify healthcare providers’ barriers to implementing the pressure injury prevention protocol. These questions were adapted by reviewing different pieces of literature (1, 27, 28).

### Statistical analysis

The authors used quantitative data analysis using IBM Corporation’s Statistical Package for the Social Sciences (SPSS) version 26.0. After data collection, all questionnaires were reviewed to ensure clarity and completeness. Subsequently, the collected data underwent descriptive analyses based on the level of measurement. Each variable under study was clearly labeled and distinguished within a single SPSS file, accompanied by detailed documentation including variable names, coding values, and other relevant information. Before conducting any analysis, the dataset was screened for the normality of distribution, presence of missing data, and identification of outliers. Descriptive statistics, such as the average (M) and the standard deviation (SD), were used to measure the total scores, and a linear multiple regression model was used to determine which factors affect the knowledge and attitudes toward the prevention of PI at the medical ward in Jordanian hospitals.

A knowledge score for each case was calculated as the total number of correct answers out of the 18 items that measure the knowledge of PI prevention. The knowledge scores range from 0 to 18; a higher score, close to 18, is considered good knowledge. To calculate the attitude scores, numerical values were given for each attitude question: 1 for “strongly disagree,” 2 for “disagree,” 3 for “neither agree nor disagree,” 4 for “agree,” and 5 for “strongly agree.” The authors calculated the attitude score as the total points received for the 11 attitude answers and ranged from 11 to 55. Similarly, the perceived barrier score was calculated as the total number of questions that were answered as “Yes” by the respondents and ranged from 0 to 11, corresponding to the 11 barrier items presented in the questionnaire.

### Ethical considerations

Ethical approval was obtained from the Ministry of Health through the Institutional Review Board (IRB no. mba/ethical committee/12724) and from the Applied Science Private University (IRB no. 2023-2024-32). The invitation for participation extends to all personnel working in the hospitals included in the study, accompanied by a detailed explanation of the research objectives, significance, and duration. Upon receiving this information, participants were requested to sign an informed consent form affirming their voluntary participation and the right to withdraw from the study at any time. To ensure confidentiality and anonymity, participants were instructed not to disclose personal details such as their names, contact information, or identification documents. All collected data were securely stored in a locked cabinet, accessible only to the researchers.

## Results

### Demographic characteristics of the sample

Table 1 shows the demographic data for physicians, which includes 49.3% women and 50.7% men. The majority of the physicians are between the ages of 30 and 39 accounting for 74% (54 physicians). In total, 69.9% are married, and more than half receive a salary of less than JMD$ 1,000 (60%). However, nearly 68% of the physicians have less than 5 years of experience and do not receive training on PI prevention (83.6%), but 69.9% used the PI guidelines. Additionally, 41 physicians (56.2%) reported an unproportionate provider-to-patient ratio.

**Table 1:** Sociodemographic characteristics of participants (*n* = 73).

Variable	*n*	%
Sex		
Male	37	50.7
Female	36	49.3
Age		
20–29	9	12.3
30–39	54	74
40–49	7	9.6
>50	0	0
Marital		
Married	51	69.9
Single	22	30.1
Divorced	0	0
Monthly income (Younis et al.)		
≤1,000 JMD	44	60.2
>1,000 JMD	29	39.8
Experience (Pannick et al.)		
≤5	25	34.3
>5	48	65.7
Received training		
Yes	12	16.4
No	61	83.6
Training date		
No	71	97.3
Old	0	0
Recent	2	2.7
Using guidelines		
Yes	51	69.9
No	22	30.1
Unproportioned providers		
Yes	41	56.2
No	32	43.8

### Knowledge of the PI prevention

Table 2 shows that the majority of physicians have a good level of knowledge regarding PI prevention because the majority of physicians chose “true” for all statements, with the frequency percentages ranging between 58.9 and 98.6%. Moreover, the highest frequency was observed for Statement 1, with approximately 72 (98.6%) physicians. This finding indicates that physicians showed a strong agreement that the risk factors for PI development are immobility, incontinence, impaired nutrition, and altered level of consciousness. In contrast, the lowest frequency was recorded for Statement 6, with only 43 (58.9%) physicians estimated to be correct. This finding suggests that fewer physicians agreed that all individuals should be assessed on admission to a hospital for the risk of PI development.

**Table 2:** Physician’s knowledge toward preventing PI (*n* = 73).

Variables	Physician’s knowledge		
	True *n* (%)	False *n* (%)	Do not Know *n* (%)
Risk factors for the development of PI are immobility, incontinence, impaired nutrition, and altered level of consciousness	72 (98.6)	1 (1.4)	0 (0)
All hospitalized individuals at risk for PI should have a systematic skin inspection at least daily, and those in long-term care at least once a week.	60 (82.8)	6 (8.2)	7 (9.6)
The first sign of PI development is redness or an open sore.	67 (91.8)	5 (6.8)	1 (1.4)
Hot water and soap may dry the skin and increase the risk of PI.	50 (68.5)	18 (24.7)	5 (6.8)
It is important to massage bony prominences.	49 (67.1)	15 (20.5)	9 (12.3)
All individuals should be assessed on admission to a hospital for the risk of PI development.	43 (58.9)	30 (41.1)	0 (0)
Patient skin should be clean and dry to prevent the risk of PI development.	64 (87.7)	9 (12.3)	0 (0)
Adequate dietary intake of protein and calories should be maintained during illness.	72 (98.6)	1 (1.4)	0 (0)
Vitamin C and E are important to maintain skin integrity.	70 (95.9)	2 (2.7)	1 (1.4)
Persons confined to bed should be repositioned every 3 h.	59 (80.8)	7 (9.6)	7 (9.6)
A person who cannot move him or herself should be repositioned every 2 h while sitting in a chair.	55 (75.3)	8 (11.0)	10 (13.7)
Heel injury is prevented by Putting a pillow under the patient’s leg.	60 (82.2)	7 (9.6)	6 (8.2)
Friction may occur when moving a person up in bed.	64 (87.7)	6 (8.2)	3 (4.1)
For persons who have incontinence, skin cleaning should occur at the time of soiling and at routine intervals.	70 (95.9)	2 (2.7)	1 (1.4)
Educational programs may reduce the incidence of Pressure Injury.	71 (97.3)	2 (2.7)	0 (0)
Stage II Pressure Injury may be extremely painful due to exposure of nerve endings.	65 (89.0)	4 (5.5)	4 (5.5)
Shear is the force that occurs when the skin sticks to the surface and the body slides.	63 (86.3)	3 (4.1)	7 (9.6)
All care given to prevent or treat PI must be documented.	66 (90.4)	3 (4.1)	4 (5.5)
**Knowledge score** **(0–18) (M ± SD)** ^*^	**(15.34 ± 1.75)**		

* M, mean; SD, standard deviation.

### Attitudes toward PI prevention

Table 3 presents the physicians’ attitudes toward PI prevention among patients in medical wards of Jordanian hospitals. Half of them (52.1–50.7%) agreed with statements one, two, and three (see Table 4). However, they should have disagreed with these statements, as they reflect a weak attitude toward PI prevention regarding universal risk awareness, prevention time demands, and treatment over prevention. The majority of physicians agreed or were unsure (neither agree nor disagree) with the rest of the statements. However, the physicians should have disagreed with statements numbers 9, 10, and 11; instead, many agreed with these statements. This indicates that they have a weak attitude toward the lack of concern, decreased PI occurrence, and belief that clinical judgment is better than the available PI risk assessment tools. This reflects a weak level of attitude among physicians.

**Table 3:** Physician’s attitude toward preventing PI (*n* = 73).

Variable	Strongly disagree *n* (%)	Disagree *n* (%)	Neither agree nor disagree *n* (%)	Agree *n* (%)	Strongly agree *n* (%)
All patients are at potential risk of developing PI.	4 (5.5)	7 (9.6)	11 (15.1)	38 (52.1)	13 (17.8)
PI prevention is time-consuming for me to carry out.	3 (4.1)	7 (9.6)	9 (12.3)	37 (50.7)	17 (23.3)
Pressure injury treatment is a greater priority than pressure injury prevention.	6 (8.2)	6 (8.2)	8 (11.0)	38 (52.1)	15 (20.5)
Continuous assessment of patients will give an accurate account of their PI risk.	2 (2.7)	6 (8.2)	15 (20.5)	34 (46.6)	16 (21.9)
The majority of PI can be avoided.	1 (1.4)	6 (8.2)	23 (31.5)	29 (39.5)	14 (19.2)
In comparison with other areas of care, PI prevention is a low priority for me.	7 (9.6)	4 (5.5)	18 (24.7)	33 (45.2)	11 (15.1)
PI risk assessment should be regularly carried out on all patients during their stay in the hospital.	1 (1.4)	6 (8.2)	20 (27.4)	35 (47.9)	11 (15.1)
I do not need to concern myself with pressure injury prevention in my practice.	8 (11.0)	14 (19.2)	17 (23.3)	29 (39.7)	5 (6.8)
I am less interested in PI prevention than in other aspects of care.	7 (9.6)	8 (11.0)	25 (34.2)	27 (37.0)	6 (8.2)
In my opinion, patients tend not to get as many PI nowadays.	3 (4.1)	13 (17.8)	31 (42.5)	20 (27.4)	6 (8.2)
My clinical judgment is better than any PI risk assessment tool available to me.	2 (2.7)	5 (6.8)	29 (39.7)	26 (35.6)	11 (15.1)
**Attitude score** **(5–55) (M ± SD)** ^*^	**(32.73 ± 4.91)**				

* M, mean; SD, standard deviation.

**Table 4:** Physician’s perceived barriers to preventing PI (*n* = 73).

Barriers	Physician’s perceived barriers	
	Yes *n* (%)	No *n* (%)
Poor access to literature and reading facilities.	62 (84.9)	11 (15.1)
Heavy workload and inadequate staff.	69 (94.5)	4 (5.5)
Lack of universal guidelines on the prevention of PI.	62 (84.9)	11 (15.1)
Inadequate training coverage of pressure injury prevention.	65 (89.0)	8 (11.0)
Uncooperative patients.	64 (87.7)	9 (12.3)
Lack of job satisfaction in your profession.	62 (84.9)	11 (15.1)
Presence of other priorities than pressure injury.	64 (87.7)	9 (12.3)
Shortage of resources (equipment/resources).	58 (79.5)	15 (20.5)
Inadequate knowledge about PI among healthcare providers.	66 (90.4)	7 (9.6)
Lack of multidisciplinary teamwork among the healthcare team.	67 (91.8)	6 (8.2)
I do not have any challenges.	56 (76.7)	17 (23.3)
**Perceived barriers score** **(0–11) (M ± SD)** ^*^	**(9.52 ± 1.81)**	

* M, mean; SD, standard deviation.

### Barriers to PI prevention

The majority of physicians answered “yes” to all statements regarding perceived barriers toward PI prevention in the medical wards of Jordanian hospitals, with agreement rates ranging between 76.7 and 94.5% (see Table 4). This finding indicates a high level of agreement on the existence of perceived barriers toward PI prevention among physicians. Nearly all participants, 69 (94.5%), identified the heavy workload and inadequate staff, followed by a lack of multidisciplinary approach among healthcare teams (91.8%) and inadequate knowledge about PI among healthcare providers (90.4%). However, the least perceived barrier toward PI prevention is the shortage of resources (equipment/resources), with 79.5%. This means that 20.5% of physicians do not consider it a perceived barrier (Table 4).

### Physicians’ knowledge, attitudes, and barriers-related factors

Table 5 presents the negative correlation between physicians’ knowledge of PI and attitudes toward PI prevention, indicated by an R-value of −0.24, which is statistically significant with a *p*-value of 0.04. This finding suggests that, with the increase in knowledge regarding pressure injury prevention, the positive attitudes of physicians may decrease. This finding could imply that higher levels of knowledge and information might lead to a more critical or realistic view of the challenges associated with PI prevention, potentially affecting their attitudes. However, the correlation between knowledge scores and perceived barriers is positive (*R* = 0.33) and shows high significance (*p* = 0.00), which indicates that, as knowledge increases, the perceived barriers to effective PI prevention also increase, suggesting that healthcare professionals who possess better knowledge regarding PI prevention are more likely to recognize and acknowledge the barriers that exist in practice.

**Table 5:** Correlation between knowledge, attitude, and perceived barriers to PI prevention.

Variables	Knowledge about PI prevention
Attitude	
*R*	−0.24*
*p* value	**0.04**
Perceived barrier	
*R*	0.33**
*p*-value	**0.00**

*Correlation is significant at *p* < 0.05. **Correlation is significant at *p* < 0.01.

Table 6 shows the relationship between the knowledge, attitudes, and perceived barriers scores and the demographic characteristics. A significant difference is shown in knowledge scores between those earning ≤JMD 1,000 (14.77) and those earning >JMD 1,000 (16.21), with a t-test value of 3.72 and a *p*-value of <0.00, indicating a strong correlation between higher income and increased knowledge. Attitude scores also differ significantly, with a mean of 33.64 for lower income and 31.34 for higher income (*p* = 0.05). The perceived barrier scores show a significant difference as well, with lower-income individuals reporting a mean of 9.16 compared to 10.07 for higher-income individuals (*p* = 0.035). However, the mean knowledge scores for those with ≤5 years of experience (14.84) and those with >5 years (15.6) show no significant difference (*p* = 0.08). Attitude scores are also similar, with a mean of 33.4 and 32.38, respectively (*p* = 0.40). The perceived barrier scores are comparable as well, with no significant differences. On the other hand, a significant difference was found between those who use guidelines compared to those who do not use guidelines (14.73), (*p* = 0.048). Those who used guidelines had a higher mean knowledge score (15.61) compared to those who did not use them. Attitude scores are slightly lower for those using guidelines (32.22) compared to those not using them (33.91), but this difference was not significant (*p* = 0.18). The perceived barrier scores also show significant differences, with those using guidelines reporting a mean of 9.82 compared to a mean of 8.82 for those not using them (*p* = 0.03).

**Table 6:** Comparing knowledge, attitude, and perceived barrier scores results by demographic information.

Demographic information	Knowledge			Attitude			Perceived barriers		
	Mean	Test	P	Mean	Test	P	Mean	Test	P
Gender Male (n = 37) Female (n = 36)	15.43 15.25	t=0.44	.66	32.0 33.47	t=1.29	.20	9.24 9.81	t=1.34	.19
Marital Status Single (22) Married (51)	15.33 15.36	t=0.07	.95	32.0 34.4	t=1.96	.054	9.63 9.27	t=0.77	.45
Age 20-29 (n=9) 30-39 (n=54) ≥ 40 (n=31)	14.22 15.35 16.14	F=2.62	.08	34.0 32.7 31.57	F=0.47	.63	10.0 9.33 9.71	F=0.59	.56
Monthly Income ≤ 1000 JD (n = 44) > 1000 JD (n=29)	**14.77** **16.21**	**t=3.72**	.00**	**33.64** **31.34**	**t=1.99**	.05*	**9.16** **10.07**	**t=2.15**	.035*
Work Experience ≤ 5 yr (n= 25) > 5 yr (n = 48)	14.84 15.6	t=1.79	.08	33.4 32.38	t=0.85	.40	9.32 9.63	t=0.68	.49
Training Yes (n =12) No (n= 61)	15.83 15.25	t=1.06	.29	33.0 32.67	t=0.21	.83	9.58 9.51	t=0.13	.89
Guidelines Yes (n =51) No (n = 22)	15.61 14.73	t=2.01	.048*	32.22 33.91	t=1.36	.18	9.82 8.82	t=2.24	.03*
Unproportioned Providers ratio Yes (n=41) No (n=32)	15.66 14.94	t=1.77	.08	32.37 33.19	t=0.71	.48	9.90 9.03	t=2.09	.041*

* Significant at *P* < 0.05, ** Significant at *P* < 0.01.

A significant difference was found in the perceived barrier scores between those who reported unproportioned provider-to-provider ratios, with a mean of 9.90, compared to those who reported proportioned ratios, with a mean of 9.03 (*p* = 0.041). Other demographic variables were not significantly different.

## Discussion

The current study aims to assess physicians regarding the level of knowledge, attitudes, and barriers toward PI prevention in general medical wards. The study shows that the physicians have good knowledge in this regard, but weak attitudes. This finding was consistent with a study conducted in a rehabilitation center in Saudi Arabia (29), where the physicians had good knowledge and weaker attitudes compared to the other healthcare professions. Rehabilitation centers receive chronic medical diseases, which are very similar to the medical wards in Jordan; however, this result contradicts the study of Hamdan et al. (20), who found that physicians’ attitudes toward PI prevention in the cancer unit in Saudi Arabia were highly positive. Our study also shows that physicians perceive high barriers in terms of PI prevention in the medical wards. Moreover, several previous studies discussed the healthcare providers as one unit of the study or focused on nurses working in special care ward units. The recognized barriers mentioned by the physicians were highly similar to the study of Sham et al. (8) among the nurses, and the physicians highly agreed that the lack of staff, high workload, and competing priorities are the main barriers to PI prevention. However, the results of the current study disagree that a lack of necessary equipment and facilities is one of the fundamental barriers. Particularly for the physicians, this result could be explained by the fact that greater knowledge about PI leads to higher expectations regarding management and working conditions similar to the guidelines; therefore, recognizing the barriers is apparent. Moreover, this finding may contribute toward a negative attitude, particularly in the medical ward, where there is a shortage of staff and pronounced limited resources. In addition to the highly demanding patient care, this study was consistent with the study of Hamdan et al. (20). Moreover, our results align with those of Azhar et al. (30), who reported a higher score of the perceived barriers regarding PI prevention, including the critical care unit assessment, inadequate workers, and ward overcrowding. In addition, this finding is further supported by the recent study conducted by Mkoka and Andwilile (31).

Physicians may face barriers that harm their attitude. Focusing on other clinical priorities, high workloads, and specific time for direct PI patient care connected to PI prevention can decrease their attention to PI. In addition, the PI patient’s cooperation and support from the patient’s family members may be inadequate, resulting in less perceived effectiveness and frustration with effective PI prevention efforts. These outcomes align with perceived barriers found by Hamdan et al. (20), who discovered negative attitudes among physicians and nurses in similar settings because of environmental and systemic challenges.

Our study highlighted significant differences in knowledge, attitudes, and perceived barriers across various demographic factors, particularly emphasizing the impact of monthly income and the use of guidelines. Specifically, those with higher incomes perceived more barriers and had less positive attitudes compared to those who received lower incomes. The reason for this could be that physicians with high incomes work in more resource-rich environments, where expectations for patient care, including PI prevention, are higher. This suggests who are more aware of organizational and systematic shortcomings are more likely to identify greater barriers. In addition, higher-income healthcare providers may have access to better learning resources, professional development opportunities, and training programs, which would provide them with more knowledge regarding PI prevention. This finding is consistent with many previous studies (13, 21, 32–34).

According to the use of guidelines, the study found that the frequent use of evidence-based guidelines leads to better practice, which is fundamentally connected to the developed knowledge. Physicians who commonly consult and use these guidelines are sufficiently knowledgeable to stay updated with the recent standards in PI prevention. It can also be indicated that healthcare providers with a higher income might work in settings with the best institutional support, where guidelines and policies are highlighted and supported as part of their routine patient care, similar to the findings of the studies conducted by Gruber (14) and by Algunmeeyn et al. (35), respectively. On the other hand, although the Ministry of Health has one payment scale, those with higher income might have other responsibilities and tasks to carry out; therefore, their input for PI prevention might be limited. In addition, they show better knowledge that allows them to consider and understand the limitations and lack of resources; therefore, adherence to guidelines might be influenced.

Although other demographic factors were not significantly related to the physical knowledge or attitudes scores, healthcare providers with various qualifications may share similar challenges in PI-related learning during their formal education and training, which leads to a weak variation in knowledge scores according to qualification levels. This finding is supported by studies by Parisod et al. (33) and Kaddourah et al. (29), which reported that education, training, and practical experience significantly impact knowledge scores. Higher qualifications, training, and good practical experience lead to better knowledge among patients regarding PI prevention.

The medical ward faces unique challenges compared to closed intensive care units. A multidisciplinary approach is highly recommended to decrease the level of PI in medical wards. In cases where physicians perceive unproportioned staffing, care for PI patients often encounters organizational and systemic barriers, such as high workloads, limited resources, and an insufficient number of physicians relative to the number of patients. These factors, combined with work priorities, are significant barriers to effective PI prevention, as similarly observed in the study by Pannick et al. (36).

## Conclusion

To conclude, our study found that, while physicians demonstrated good knowledge of pressure injury prevention, they encountered several barriers that negatively affected their attitudes toward PI prevention in medical wards. This finding highlights the need for healthcare systems to implement practical organization, systematic training, and support to foster positive attitudes among healthcare providers. Enhancing their confidence in applying PI preventive methods, promoting a deeper understanding of patient safety, and encouraging continuous education are essential steps. As healthcare professionals gain more experience and knowledge, they become better equipped to adopt effective practices and implement policies to prevent PI, ultimately improving the quality of patient care and creating a safer healthcare environment. Furthermore, it is crucial to strengthen the multidisciplinary team approach to PI care and prevention by clarifying policies and defining roles and responsibilities among all healthcare providers. This finding will enable physicians to better understand their roles and participate more effectively in medical wards. Further research on physicians’ overall working limitations and challenges would be recommended.

## Data Availability

The raw data supporting the conclusions of this article will be made available by the authors without undue reservation.

## References

[1] Bryant RA Moore ZEH Iyer V. Clinical profile of the SEM scanner — modernizing pressure injury care pathways using sub-epidermal moisture (SEM) scanning. Expert Rev Med Devices. (2021) 18:833–47. doi: 10.1080/17434440.2021.1960505, PMID: 34338565

[2] Alanazi FK Sim J Lapkin S. Systematic review: nurses’ safety attitudes and their impact on patient outcomes in acute-care hospitals. Nurs Open. (2022) 9:30–43. doi: 10.1002/nop2.1063, PMID: 34538027 PMC8685891

[3] Isfahani P Alirezaei S Samani S Bolagh F Heydari A Sarani M. Prevalence of hospital-acquired pressure injuries in intensive care units of the eastern Mediterranean region: a systematic review and meta-analysis. Patient Saf Surg. (2024) 18:1. doi: 10.1186/s13037-023-00383-8, PMID: 38167487 PMC10763125

[4] Li J Zhu C Liu Y Li Z Sun X Bai Y. Critical care nurses’ knowledge, attitudes, and practices of pressure injury prevention in China: a multicentric cross-sectional survey. Int Wound J. (2023) 20:381–90. doi: 10.1111/iwj.13886, PMID: 35906851 PMC9885482

[5] Howell M Loera S Tickner A Maydick-Youngberg D Faust E Martin S. Practice dilemmas: conditions that mimic pressure ulcers/injuries-to be or not to be? Wound Manag Prevent. (2021) 67:12–38. doi: 10.25270/wmp.2021.2.1238 33544693

[6] Rapetti R Pansera A Visca S Martolini M Antoniotti S Bertoncini F. Pressure ulcers in hospital patients: incidence and risk factors. J Wound Care. (2023) 32:29–34. doi: 10.12968/jowc.2023.32.1.29, PMID: 36630116

[7] Bjurbo C Wetzer E Thunborg D Zhang L Hultin L. Knowledge and attitudes regarding pressure injuries among assistant nurses in a clinical context. Int Wound J. (2024) 21:e14950. doi: 10.1111/iwj.14950, PMID: 38923719 PMC11199803

[8] Sham F Sharif DIB Binti Moksin N And Selamat H. Knowledge, practice and perceived barrier of pressure ulcer prevention among nurses in a public hospital in Selangor. Malay J Public Health Med. (2020) 20:325–35. doi: 10.37268/mjphm/vol.20/no.Special1/art.738

[9] Ghazanfari MJ Karkhah S Maroufizadeh S Fast O Jafaraghaee F Gholampour MH. Knowledge, attitude, and practice of Iranian critical care nurses related to prevention of pressure ulcers: a multicenter cross-sectional study. J Tissue Viability. (2022) 31:326–31. doi: 10.1016/j.jtv.2022.01.009, PMID: 35115222

[10] Sari Y Upoyo AS Mulyono WA Sumeru A Taufik A Nuriya N. Pressure injury prevention: knowledge and attitude and their predictors in Indonesian nurses working in hospital settings. J Tissue Viability. (2023) 32:242–7. doi: 10.1016/j.jtv.2023.04.002, PMID: 37037689

[11] Ledger L Worsley P Hope J Schoonhoven L. Patient involvement in pressure ulcer prevention and adherence to prevention strategies: an integrative review. Int J Nurs Stud. (2020) 101:103449. doi: 10.1016/j.ijnurstu.2019.103449, PMID: 31706155

[12] Berihu H Wubayehu T Teklu T Zeru T Gerensea H. Practice on pressure ulcer prevention among nurses in selected public hospitals, Tigray, Ethiopia. BMC Res Notes. (2020) 13:207. doi: 10.1186/s13104-020-05049-7, PMID: 32276650 PMC7149915

[13] Wu Z Song B Liu Y Zhai Y Chen S Lin F. Barriers and facilitators to pressure injury prevention in hospitals: a mixed methods systematic review. J Tissue Viability. (2023) 32:355–64. doi: 10.1016/j.jtv.2023.04.009, PMID: 37150650

[14] Gruber J. Financing health care delivery. Annu Rev Financ Econ. (2022) 14:209–29. doi: 10.1146/annurev-financial-111620-112740

[15] Zhao J Zhang L-X Zhong Y-L Hu X-Y Cheng Y Zhou Y-F. A 10-year prevalence survey and clinical features analysis of pressure injury in a tertiary hospital in China, 2009–2018. Adv Skin Wound Care. (2021) 34:150–6. doi: 10.1097/01.asw.0000732740.92841.51, PMID: 33587476

[16] Shiferaw WS Akalu TY Mulugeta H Aynalem YA. The global burden of pressure ulcers among patients with spinal cord injury: a systematic review and meta-analysis. BMC Musculoskelet Disord. (2020) 21:334. doi: 10.1186/s12891-020-03369-0, PMID: 32471497 PMC7260823

[17] Li Z Lin F Thalib L Chaboyer W. Global prevalence and incidence of pressure injuries in hospitalised adult patients: a systematic review and meta-analysis. Int J Nurs Stud. (2020) 105:103546. doi: 10.1016/j.ijnurstu.2020.103546, PMID: 32113142

[18] Younis WY Abdalrahim MS Zeilani RS Albusoul R Alosaimi D Hamdan-Mansour AM. Feasibility and clinical utility of bates-Jensen wound assessment tool among nurses caring of patients having pressure ulcers. South Eastern Eur J Public Health. (2023). doi: 10.56801/seejph.vi.229

[19] Reza HM Das CK Mittra CR Saha AK. Nurses’ knowledge and practices regarding prevention and management of pressure ulcer for hospitalized patient. Asian J Med Biol Res. (2020) 6:237–43. doi: 10.3329/ajmbr.v6i2.48055

[20] Hamdan A Duraisamy B Javison S Wani T Alharbi M. Physicians attitude towards pressure injury prevention and management. J Cancer Ther. (2020) 11:189–98. doi: 10.4236/jct.2020.114016

[21] Lee S-B Lee H-Y. Impact of pressure ulcer prevention knowledge and attitude on the care performance of long-term care facility care workers: a cross-sectional multicenter study. BMC Geriatr. (2022) 22:988. doi: 10.1186/s12877-022-03702-3, PMID: 36544097 PMC9769059

[22] Levine JM Levine JM Leveille N LeBarron DJ. Physician involvement in wound care: barriers and strategies. J Am Med Dir Assoc. (2013) 14:B24–5. doi: 10.1016/j.jamda.2012.12.070

[23] LoBiondo-Wood G Haber J. Nursing research E-book: methods and critical appraisal for evidence-based practice Elsevier: Health Sciences (2021).

[24] Boltz M Capezuti E Zwicker D Fulmer TT. Evidence-based geriatric nursing protocols for best practice, Springer Publishing Company (2020).

[25] Tesfa Mengist S Abebe Geletie H Zewudie BT Mewahegn AA Terefe TF Tsegaye Amlak B. Pressure ulcer prevention knowledge, practices, and their associated factors among nurses in Gurage zone hospitals, South Ethiopia, 2021. SAGE Open Med. (2022) 10:20503121221105571. doi: 10.1177/20503121221105571, PMID: 35756351 PMC9218504

[26] Etafa W Argaw Z Gemechu E Melese B. Nurses’ attitude and perceived barriers to pressure ulcer prevention. BMC Nurs. (2018) 17:14. doi: 10.1186/s12912-018-0282-2, PMID: 29686535 PMC5902867

[27] Källman U Suserud BO. Knowledge, attitudes and practice among nursing staff concerning pressure ulcer prevention and treatment--a survey in a Swedish healthcare setting. Scand J Caring Sci. (2009) 23:334–41. doi: 10.1111/j.1471-6712.2008.00627.x 19645807

[28] Tubaishat A Aljezawi M Al Qadire M. Nurses’ attitudes and perceived barriers to pressure ulcer prevention in Jordan. J Wound Care. (2013) 22:490–7. doi: 10.12968/jowc.2013.22.9.490, PMID: 24005783

[29] Kaddourah B Abu-Shaheen AK Al-Tannir M. Knowledge and attitudes of health professionals towards pressure ulcers at a rehabilitation hospital: a cross-sectional study. BMC Nurs. (2016) 15:17. doi: 10.1186/s12912-016-0138-6, PMID: 26949373 PMC4779255

[30] Azhar SW Sharoni SKA Fauzi R Isa R Shohor NA Seman N. Knowledge, attitude and perceived barrier towards pressure ulcer prevention among critical care unit nurses in Klang Valley public hospitals. Malay J Med Health Sci. (2022) 18:56–59. doi: 10.47836/mjmhs18.8.9

[31] Mkoka DA Andwilile R. Nurses’ perceptions on barriers for implementing pressure ulcers preventive measures among critically ill patients at a tertiary teaching hospital, Tanzania. Int J Africa Nurs Sci. (2024) 20:100676. doi: 10.1016/j.ijans.2024.100676

[32] Muhammed EM Bifftu BB Temachu YZ Walle TA. Nurses’ knowledge of pressure ulcer and its associated factors at Hawassa university comprehensive specialized hospital Hawassa, Ethiopia, 2018. BMC Nurs. (2020) 19:51. doi: 10.1186/s12912-020-00446-6, PMID: 32549784 PMC7296692

[33] Parisod H Holopainen A Kielo-Viljamaa E Puukka P Beeckman D Haavisto E. Attitudes of nursing staff towards pressure ulcer prevention in primary and specialised health care: a correlational cross-sectional study. Int Wound J. (2022) 19:399–410. doi: 10.1111/iwj.13641, PMID: 34121328 PMC8762573

[34] Yoon JE Cho OH. Risk factors associated with pressure ulcers in patients with traumatic brain injury admitted to the intensive care unit. Clin Nurs Res. (2022) 31:648–55. doi: 10.1177/10547738211050489, PMID: 34622689

[35] Algunmeeyn A El-Dahiyat F Al-Hussami M. Exploring the factors that influence healthcare providers care quality in Jordanian hospitals: the perspectives of nurses, pharmacists and physicians. J Pharm Health Serv Res. (2021) 12:509–13. doi: 10.1093/jphsr/rmab035

[36] Pannick S Davis R Ashrafian H Byrne BE Beveridge I Athanasiou T. Effects of interdisciplinary team care interventions on general medical wards: a systematic review. JAMA Intern Med. (2015) 175:1288–98. doi: 10.1001/jamainternmed.2015.2421, PMID: 26076428

